# Progress of Surgery

**Published:** 1903-02-28

**Authors:** 


### PROGRESS OF SURGERY.

(*Continued from page* 341.)

**Tubercular Kidney.**—Edgar Garceau, of Boston, Mass.,[4] has considered the results of operations for tubercular kidney. For the purpose he has himself collected a list of 194 cases, and added to these the previously published cases of Bangs and Fackland, making 415 cases in all. In these three lists, taken in the order mentioned, the proportion of cures at the end of two years after the operation was, respectively, 21, 7·4, and 7·9 per cent. Thus the results have been anything but good. It is encouraging that the latest statistics are considerably better than the earlier ones, probably by reason of the later cases being more judiciously selected and operated upon at an earlier period in their evolution. The results are bad, because, according to the author, renal tuberculosis is rarely primary, but takes its origin from a primary focus elsewhere in the body, and this pre-existing disease, subsequently in many instances, causes death. It may be argued that the other foci of disease revealed during life, or at post-mortems, may have been secondary, and there is no means of proving this was not so. The question as to whether the renal lesion was the primary one or not could only be settled by autopsies in early cases of renal tuberculosis, and such are rarely obtained. In the author's collected cases tuberculosis of the opposite kidney was reported in 5·7 per cent. The lungs were most frequently affected, and undoubtedly were the starting point of the disease in many instances. Some brilliant cures were noticeable in the list, the most remarkable being one of Czerny's cases, in which cure persisted for 21 years. Such cases are very rare, and a patient who has been operated upon for renal tuberculosis should never consider that the future is safe. Such patients should take the utmost care in order to maintain their constitutions up to the highest pitch of vigour. The percentage of cures in 22 cases in which nephrotomy was performed was only ·56 per cent. In the author's cases the best results, both immediate or late, were from the operations of nephrotomy followed by nephrectomy. In 47 of these cases the mortality was 11·9 per cent., and the number of permanent cures 25·4 per cent. Probably these were cases in which a large abscess was first opened, and nephrectomy done when the patients had recuperated. The mortality after nephrectomy in the author's cases was 17·4 per cent., the best on record. Resection of a tubercular kidney is condemned by Albarran, König, and others. Ureterectomy together with nephrectomy is the ideal operation. Complete recovery followed in some cases where a diseased ureter was left. But the mortality is great if this is done, and fistula, secondary abscess, and continuance and persistence of urinary symptoms are apt to follow.

**Surgery of the Ureters.**—Young, of Baltimore,[5] has a paper on the treatment of calculus in the lower end of the ureter in the male. There is no difficulty in gaining access to the upper three-fourths of the ureter. The lower fourth, however, is not easily exposed. Without opening the peritoneum, it could be reached by the sacral operation of Kraske, but apparently this operation has never been performed. In 1899 Mr. Morris said that no case had hitherto been recorded of uretero-lithotomy for stone impacted in the lower end of the ureter in the male. Young reports three cases of the operation performed by himself and a fourth treated by Finney. In the first case the incision was made from a point above the middle of Poupart's ligament upwards and outwards, and the operation was extra-peritoneal throughout. After removal of the stone the portion of ureter below was found to be constricted, and was divided through an incision on the lateral aspect of the bladder, and the incisions in the bladder and ureter were closed with fine silk sutures. Five months later the patient was perfectly well. Reviewing the literature the author finds that 18 male patients have been treated for pelvic ureteral calculi. In nine the intra-vesical method was used, in one the perineal, in one the intra-rectal, and in seven the iliac (extra-peritoneal). Twelve of the patients recovered, three died, and in three the results are not stated. The ureter is constricted where it enters the muscular wall of the bladder and again where it opens into the bladder, and the commonest sites for calculi are at one or other of these constrictions, *e.g.* in 13 cases out of 19. The symptoms simulate those of stone in the bladder. Rectal and cystoscopic examination are most important for diagnosis, the latter because the ureteric orifice may be much changed in appearance. The Roentgen rays have several times revealed the stone. When the calculus is in the intra-vesical part of the ureter, supra-pubic cystotomy and incision of the ureter through the bladder is the proper operation ; when extra-vesical, the iliac-extra-peritoneal operation is the best. If a stricture is found also it must be dilated or divided. The incision permits of a thorough examination of the kidney and of the ureter throughout its whole extent.

W. E. Morgan, of Chicago,[6] has reported a case in which a ureter was exposed for obstruction due to a valve-like formation in its lower portion. After three long and difficult operations the obstruction was finally relieved. The patient was a man of 31 years of age, and the symptoms had existed with intermissions for four years before the first operation. The symptoms were attacks of severe pain like those of renal colic and hæmaturia, and a sausage-shaped tumour, tense and fluctuating, was found extruding from the left renal region low into the pelvis. The patient was thought to be suffering from ureteral calculus. At the first operation the left kidney was examined and found to be somewhat enlarged, and then the ureter exposed to a point below the crest of the ilium. At first sight the distended ureter gave the impression that the colon was being dealt with. It was incised one inch below the iliac spine and the lower three inches of its interior explored with Kelly's specula. It appeared to end in a blind pouch below. Then the operation, having lasted three hours, had to be stopped. The ureteral wound below was sutured and the kidney drained through a second ureteral incision which had been made near the kidney. An attack of double nephritis occurred three weeks later. The second operation consisted in opening the bladder supra-pubically, and passing one sound up the left ureter and another down from the lumbar opening. The two sounds could not be made to meet. Ether was badly borne by the kidneys in this case. Five weeks later one more attempt was made to deal with the obstruction. The ureter was exposed in its lower part to the bladder and incised. By lifting up the tip of the seminal vesicle the last portion of the ureter was seen to be of normal size. The bladder was again opened and a probe passed upwards into the ureter, and a sound downwards to try and get the obstruction passed and the two instruments brought into contact. The cause of the obstruction was apparently a reduplicated transverse fold of mucous membrane, and the existence of tough connective tissue externally pinching the ureter. The fold was incised from within, and then a No. 6 gum catheter could be passed from the lumbar opening down into the bladder. The catheter was left *in situ*. The patient made an excellent recovery.

W. R. Nicholson[7] has written on the methods of treating ureters severed during abdominal operations. Until ten years ago nephrectomy was performed for such injuries, but that operation is now discarded. The best procedures are uretero-cystotomy, or uretero-ureteral anastomosis. The injury usually occurs near the base of the broad ligament. Most authors prefer the former of the two operations mentioned. To diminish tension the bladder can be freed in front or the ureter displaced into a straighter position. Van Hook's technique is followed, viz. junction of the upper end of the ureter with an opening into the bladder, made as high as possible. To facilitate sutures a ureteral catheter may be inserted. The distal end of the ureter is closed. By placing the opening in the bladder high, and by regular catheterisation after the operation, the complications of infection, fistula, stricture, and back-flow of the urine can be avoided. Kelly prefers joining the two ends of the ureter. According to circumstances, the junction may be (1) end to side, (2) transverse, (3) oblique, or (4) by invagination. Transplantation of one or both ureters into the bowel always resulted in failure, from kidney infection, uræmia, or peritonitis. Borée recommends rather transplantation of the ureters into the vagina. Even if the above operations are not practicable, nephrectomy should not be performed. The ureter should be simply ligatured, when atrophy of the kidney will usually follow, hydronephrosis being a rare sequence.

**Stone in the Kidney.**—R. Guiteras[8] has written on the diagnosis and treatment of nephrolithiasis, and reported several cases of interest. The symptoms did not always correspond in severity to the extent of the disease. In several cases the symptoms were slight although the kidneys were almost completely destroyed. For the collection of urine from one kidney the author frequently used with success the Harris segregator. If catheterisation of the ureters did not with certainty demonstrate the presence of two kidneys, the author preferred to make a preliminary abdominal exploration of the kidneys rather than to expose each kidney by the lumbar operation. Nephrectomy after a previous nephrotomy and removal of stones always proved very difficult owing to adhesions, and several times in such operations the peritoneum was opened. This did not always lead to peritonitis. One case, a man of 48, had for many years had attacks of left renal colic. Cystitis with gonococci existed. Catheterisation of the ureters showed, in contrast to the clinical symptoms, that pyelo-nephritis existed on the right side and pyetitis on the left side. The stone proved to be in the right kidney. Another man of 48 had had a lumbar fistula for two years, since the opening of a lumbar abscess. At the operation a stone was found to have made its way out of the kidney and to lie behind that organ. Only the posterior half of the kidney could be removed.

**Determination of Renal Sufficiency.**—S. P. Fedorow,[9] on the basis of an examination of eight cases, entirely supports the claims of Casper that the determination of the freezing point of urine affords valuable help in determining renal sufficiency. The writer considers, however, that it is necessary to have a catheter in each ureter, and not to rely on one catheter in a ureter and one in the bladder, because some urine will escape by the side of the ureteral catheter into the bladder. With healthy kidneys the difference in the freezing points of the urine from each kidney may differ to the extent of 0·15° C., and the difference in specific gravity to the extent of 0·05. Ten or twenty minutes seldom suffice for collecting 20 to 30 c.c. of urine from the ureters, from half an hour to two or three hours being usually required.

**Complete Excision of the Bladder.**—A. W. Mayo Robson[7] records an example of the above operation. The operation has been performed by Bardenheuer and Gassenbauer, and Paulick of Prague and Clado have each had a successful case in which the operation was done in two stages, the ureters being first transplanted and the bladder removed later. Robson's patient, a woman of 42, had undergone several operations for tumour of the bladder before the complete excision was undertaken. Thus two years before a tumour the size of an apple had been removed, and nine months later several small tumours had been removed through the dilated urethra. The bladder had been again cleared of growth twelve months later. For the removal of the bladder a vertical incision 4 inches in length was made in the middle line of the abdomen, and a transverse incision 3 inches in length immediately above the pubes. The peritoneum was opened. The bladder formed almost a solid mass. The organ was gradually stripped from its peritoneal, uterine and vaginal attachments. The ureters were exposed close to their entrance into the bladder. The urethra was the last portion cut across. A great many small vessels required ligature. No. 6 Jaques's catheters were inserted into the divided ureters and secured by stitches, and then passed into the vagina through small incisions on each side and secured there by two or three stitches. From the first the urine passed from the left ureter was offensive, bloody, and scanty. On the thirteenth day the patient died in a uræmic condition. Had the patient recovered it was Robson's intention to try and convert the vagina into a pseudo-bladder, and to make use of the urethra which had been left *in situ*.

[4] Annals of Surg., Oct. 1902, p. 515. [5] Abst. Scottish Med. and Surg. Jour., Nov. 1902, p. 457. [6] Annals of Surg., Oct. 1902, p. 528. [7] Abst. Centralb. f. Chir., Oct. 25, 1902, p. 1115. [8] Ibid., Aug. 9, 1902, p. 862. [9] Ibid., Sept. 6, 1902, p. 944. [10] Brit. Med. Jour., Nov. 8, 1902, p. 1519.

